# Ethanol Ablation Therapy Drives Immune-Mediated Antitumor Effects in Murine Breast Cancer Models

**DOI:** 10.3390/cancers14194669

**Published:** 2022-09-25

**Authors:** Corrine A. Nief, Adam M. Swartz, Erika Chelales, Lauren Y. Sheu, Brian T. Crouch, Nirmala Ramanujam, Smita K. Nair

**Affiliations:** 1 Department of Biomedical Engineering, Duke University, Durham, NC 27708, USA; 2 Stanford School of Medicine, Stanford University, Stanford, CA 94305, USA; 3 Department of Surgery, Duke University School of Medicine, Durham, NC 27708, USA; 4 Department of Pharmacology and Cancer Biology, Duke University, Durham, NC 27708, USA; 5 Duke Global Health Institute, Duke University, Durham, NC 27708, USA; 6 Department of Pathology, Duke University School of Medicine, Durham, NC 27708, USA; 7 Department of Neurosurgery, Duke University School of Medicine, Durham, NC 27708, USA

**Keywords:** breast cancer, myeloid derived supressor cells, immunomodulation, tumor ablation, low-resource settings, tumor microenvironment

## Abstract

**Simple Summary:**

Tumor ablation is the process of directly destroying tumor tissue by injecting a cytotoxic substance, in this case, ethanol ethylcellulose. In this report, we characterized the effect of ablation on local and systemic immunologic markers known to impact disease progression in several mouse models. Ablation improved overall survival in poorly invasive breast cancer models and was notable for demonstrating an increase in tumor infiltrating lymphocytes. However, in a metastatic breast cancer model, the response to ablation was more nuanced: the growth of the primary tumor was only modestly slowed compared to controls, and there was a reduction in pro-tumor granulocytic myeloid derived suppressor cells (gMDSCs) with a reduction in metastatic disease. A single ablation reduced circulating granulocytic colony stimulating factor, tumoral gMDSCs, splenic gMDSCs, and pulmonary gMDSCs, as well as the suppressive ability of MDSCs on CD4 and CD8 T cells. The immunomodulation incited by tumor ablation was utilized to recover response to checkpoint inhibitors, resulting in increased overall survival compared to checkpoint inhibitors alone, demonstrating a proof-of-concept for using ethanol ablation as an adjuvant immunomodulatory therapy.

**Abstract:**

Ethanol ablation is a minimally invasive, cost-effective method of destroying tumor tissue through an intratumoral injection of high concentrations of cytotoxic alcohol. Ethyl-cellulose ethanol (ECE) ablation, a modified version of ethanol ablation, contains the phase-changing polysaccharide ethyl-cellulose to reduce ethanol leakage away from the tumor. Ablation produces tissue necrosis and initiates a wound healing process; however, the characteristic of the immunologic events after ECE ablation of tumors has yet to be explored. Models of triple-negative breast cancer (TNBC), which are classically immunosuppressive and difficult to treat clinically, were used to characterize the immunophenotypic changes after ECE ablation. In poorly invasive TNBC rodent models, the injury to the tumor induced by ECE increased tumor infiltrating lymphocytes (TILs) and reduced tumor growth. In a metastatic TNBC model (4T1), TILs did not increase after ECE ablation, though lung metastases were reduced. 4T1 tumors secrete high levels of granulocytic colony stimulating factor (G-CSF), which induces a suppressive milieu of granulocytic myeloid-derived suppressor cells (gMDSCs) aiding in the formation of metastases and suppression of antitumor immunity. We found that a single intratumoral injection of ECE normalized tumor-induced myeloid changes: reducing serum G-CSF and gMDSC populations. ECE also dampened the suppressive strength of gMDSC on CD4 and CD8 cell proliferation, which are crucial for anti-tumor immunity. To demonstrate the utility of these findings, ECE ablation was administered before checkpoint inhibitor (CPI) therapy in the 4T1 model and was found to significantly increase survival compared to a control of saline and CPI. Sixty days after tumor implant no primary tumors or metastatic lung lesions were found in 6/10 mice treated with CPI plus ECE, compared to 1/10 with ECE alone and 0/10 with CPI and saline.

## 1. Introduction

Over half a million people die from breast cancer each year, the most-diagnosed type of cancer globally [1]. Breast cancers that express estrogen receptors (ER), progesterone receptors (PR), or human epidermal growth factor receptor 2 (HER2) can be treated with targeted therapies; however, approximately 15% of breast cancers are triple-negative breast cancer (TNBC), lacking significant expression of hormone and growth factor receptors. The prognosis for TNBC is worse than other breast cancer subtypes because they are aggressive, invasive, high grade, and currently have no available targeted therapies [2]. Risk factors for TNBC include age, obesity, high breast density, positive BRCA mutation status, and African American ethnicity [3,4,5].

Cancer patients in low-resource areas with limited access to screening, diagnostic, and treatment options die more frequently from their cancer. Surgery and radiation, which are crucial for control of non-metastatic cancer, require extensive medical infrastructure and are inaccessible for many patients globally [6]. A developing technology that could be used when surgery and radiation are cost-prohibitive is ablation, specifically ethylcellulose-ethanol ablation (ECE). ECE consists of two components, ethanol and ethyl-cellulose, which can be injected directly into a lesion. Ethanol functions as the cytotoxic agent and the ethyl-cellulose polymer undergoes a liquid-to-solid phase change when it contacts aqueous tissue to create a slow-release depot of ethanol at the injection site [7,8]. ECE ablation produces more predictable ablation zones and less off-target damage than ablation with ethanol alone [7,9]. ECE ablation decreased rates of tumor growth [7,10,11,12] and protected against tumor development [13,14] in several preclinical animal models.

We have previously shown that intratumoral injection with ethyl cellulose-ethanol (ECE) promotes antitumor benefits against solid tumors in animal models, in part, through the direct destruction of the tumor mass [7,15]. Upon ECE injection into the tumor, ethanol induces coagulative necrosis via cytoplasmic dehydration and protein denaturation [16]. In this study, we examined whether the antitumor effects of ECE therapy had an immunological component, given that tumor necrosis facilitates secondary immunological antitumor benefits [17,18]. We assessed the immunomodulatory properties of ECE ablation using several murine models of breast cancer, including a 7,12-dimethylbenz[a]anthracene (DMBA) induced rat model, 67NR mouse model, and 4T1 mouse model. Both the 67NR mouse model and the DMBA rat model have been described as non-metastatic [19,20], while the 4T1 model is aggressively metastatic, with measurable metastases in the lungs forming 11.5 days after implantation on average [21]. This work is the first characterization of the immunologic changes induced by the ablation of orthotopic breast tumors with ECE, a low-cost minimally invasive therapy. ECE ablation was found to significantly increase tumor infiltrating T cell populations in the 67NR and DMBA induced model and reduce suppressive myeloid cell populations in the metastatic 4T1 model. Notably, a single treatment with ECE led to increased survival times in the 67NR model and a reduction in lung metastases in the 4T1 model. Better understanding of these mechanisms will enable improved implementation of ECE therapy which could help improve access to cancer care in both low- and high-resource settings.

## 2. Results

### 2.1. ECE Promotes CD8+ T Cell-Dependent Antitumor Effects in Poorly Invasive TNBC Models

**Figure 1:**
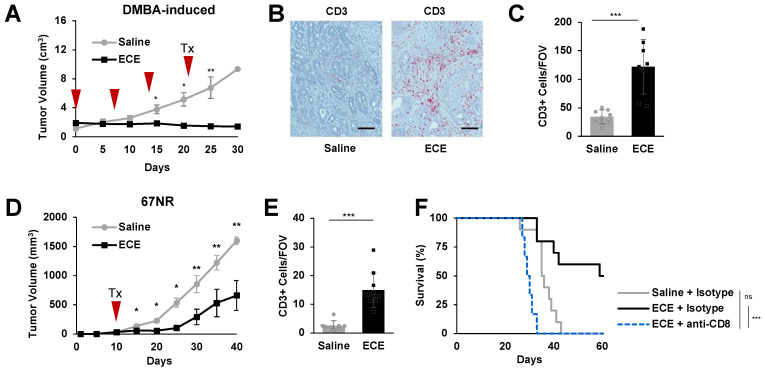
ECE promotes enhanced T cell infiltration and CD8+ T cell-dependent antitumor effects in poorly invasive breast cancer models. (**A**) Tumor growth for rats with DMBA-induced tumors receiving either ECE or saline intratumoral injections, *n* = 8. (**B**) Representative images of CD3 staining in DMBA-induced tumors collected at day 30. (**C**) Average CD3+ cells per field of view (FOV), *n* = 8. (**D**) Tumor growth for mice with 67NR tumors, *n* = 10. (**E**) Average CD3+ cells per FOV. (**F**) Kaplan–Meier survival curves of mice receiving either ECE or saline intratumoral injections with CD8 depletion or isotype controls, *n* = 10. * *p* < 0.05, ** *p* < 0.01, *** *p* < 0.001.

The effects of ECE were first evaluated against rat breast tumors generated with the potent carcinogenic polycyclic aromatic hydrocarbon, 7,12-dimethylbenz[a]anthracene (DMBA), which results in the development of poorly invasive mammary carcinomas in 100% of Sprague Dawley rats within 2 months after DMBA treatment [22]. Mammary pads of the rats were monitored twice weekly until a tumor nodule was palpable (approximately 1 cm^3^), after which the rats were randomized to receive either 200 µL of ECE or saline control administered intratumorally. As the rat mammary tumors could not be detected until they are larger than a single injection volume, treatment was given weekly: on study days 0, 7, 14, and 21. On study day 30 the tumors were harvested for immunohistochemistry. Interestingly, ECE promoted significant antitumor effects against these tumors (Figure 1A), leaving only small palpations that contained higher proportions of CD3+ T cells compared to saline treated controls at day 30 (Figure 1B,C).

Next, we assessed intratumoral ECE therapy in the poorly metastatic 67NR mouse mammary carcinoma model. Treatment of small orthotopic 67NR tumors with ECE 10 days after tumor implantation (with tumors approximately 50 mm^3^ in volume) led to a significant antitumor benefit. As with DMBA-induced rat breast tumors, we observed a significant increase in the density of CD3+ T cells compared to saline treated controls (Figure 1E, Supplementary Figure S1A). Depletion of CD8+ T cells abrogated the antitumor benefit of ECE therapy, indicating the necessity of this immune cell subset in the therapeutic response (Figure 1F). Collectively, these results suggest that intratumoral therapy with ECE can promote CD8+ T cell-dependent antitumor effects against poorly invasive breast cancer models.

### 2.2. ECE Modulates the Myeloid Compartment in Mice Bearing Highly Invasive 4T1 Tumors

**Figure 2:**
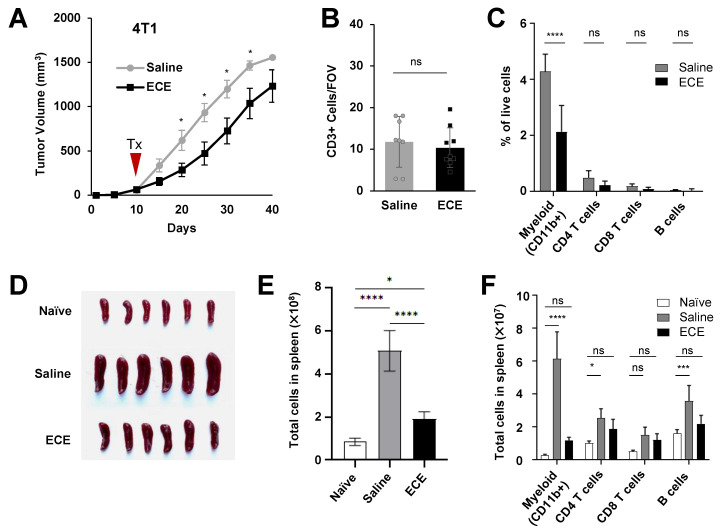
ECE does not alter the lymphoid compartment but suppresses the myeloid compartment in mice bearing highly invasive 4T1 tumors. (**A**) 4T1 tumor growth curve. Mice (*n* = 10) were treated i.t. on day 10 with saline or ECE. (**B**) Histological CD3+ cell counts (*n* = 8) and (**C**) myeloid and lymphoid immune cells, based on flow cytometry, in 4T1 tumors 7 days after i.t. saline or ECE treatment (*n* = 5). (**D**) Spleen sizes (*n* = 6), (**E**) splenocyte counts (*n* = 6), and (**F**) spleen-derived myeloid and lymphoid immune cells from tumor naïve BALB/c mice or treated 4T1 tumor-bearing mice (*n* = 5). i.t. = intratumoral(ly) * *p* < 0.05, *** *p* < 0.001, **** *p* < 0.0001.

Roughly 15–50% of all patients with TNBC develop metastatic diseass—a cohort that experiences worse survival outcomes compared to their non-metastatic counterpart [23,24]. We, therefore, examined the effects of ECE therapy in the context of the highly invasive and metastatic mouse TNBC model, 4T1. Despite the aggressiveness of 4T1 tumors, we observed a modest antitumor benefit upon treatment with ECE 10 days following orthotopic tumor implantation (Figure 2A); however, unlike the prior DMBA-induced and 67NR breast cancer models, an increased proportion of intratumoral CD3+ T cells was not detected (Figure 2B, Supplementary Figure S1B).

To gain a comprehensive understanding of the immunological changes associated with ECE therapy, we used flow cytometry to profile the major immune cell populations within tumors 7 days after ECE treatment to allow for sufficient wound healing processes to take place. No significant differences in the proportion of CD8+ T cells, CD4+ T cells, or B cells was observed among the ECE-treated and saline-treated cohorts; however, a significant decrease was observed in the proportion of CD11b+ myeloid cells in the ECE-treated group (Figure 2C). 4T1 tumors induce prominent splenomegaly, therefore, spleens were excised and analyzed 7 days after treatment. Spleen sizes were affected by ECE therapy, indicating a systemic immunological effect (Figure 2D). A single dose of ECE was capable of decreasing spleen size and total cell numbers similar to that of spleens from tumor-free mice (Figure 2D,E). An examination of immune cell subsets within spleens revealed that saline-treated mice bearing 4T1 tumors possessed slight increases in the number of CD4+ T cells and B cells and a dramatic increase in CD11b+ myeloid cells, compared to tumor naïve mice. Single-dose ECE therapy was able to resolve these alterations (Figure 2F).

### 2.3. ECE Therapy Suppresses Granulocytic Myeloid-Derived Suppressor Cells (MDSCs) in 4T1 Tumor-Bearing Mice

**Figure 3:**
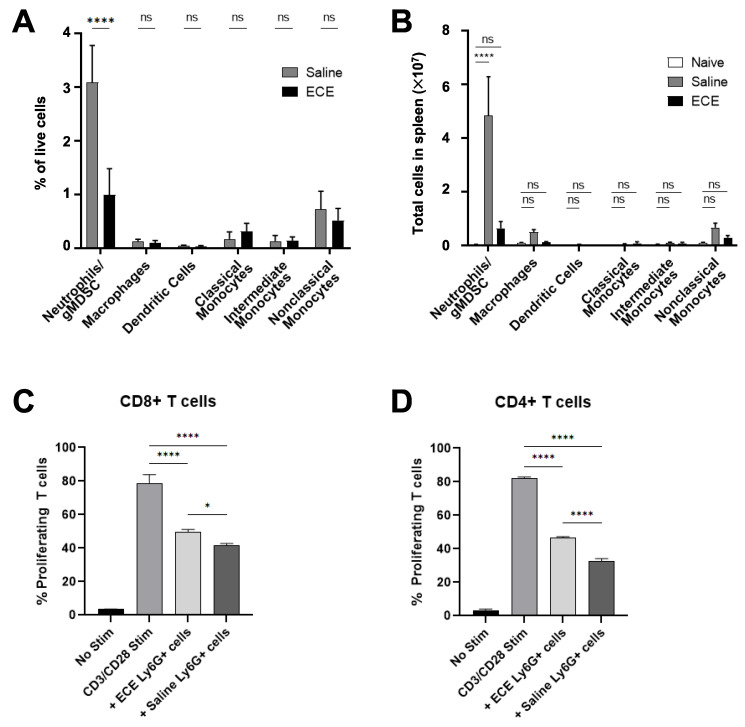
ECE reduces number and suppressive capabilities of gMDSCs in 4T1 tumor-bearing mice. Myeloid immune cell subsets within (**A**) 4T1 tumors (*n* = 5) and (**B**) spleens (*n* = 6) 7 days following i.t. saline or ECE therapy, as determined by flow cytometry. Spleens from tumor naïve mice used as controls. Proliferation of anti-CD3/CD28 stimulated (**C**) CD8+ and (**D**) CD4+ T cells in the presence of Ly6G+ cells from i.t. saline- or ECE-treated 4T1-bearing mice. i.t. = intratumoral. * *p* < 0.05, **** *p* < 0.0001.

To further characterize the changes within the myeloid compartment following treatment with intratumoral ECE therapy, we assessed levels of major myeloid cells subsets by flow cytometry 7 days following therapy using the gating strategy outlined in Supplementary Figure S2. ECE treatment promoted a ~3-fold reduction in CD45+CD11b+Ly6C+Ly6G+ neutrophils in 4T1 tumors and >5-fold reduction of these cells in the spleens of 4T1 tumor-bearing mice (Figure 3A,B). No changes in any other myeloid cell subsets were detected. Previous studies have shown that CD11b+Ly6C+Ly6G+ cells in 4T1 tumor-bearing mice represent granulocytic myeloid-derived suppressor cells (gMDSCs) that have several immunosuppressive and pro-tumorigenic functions, such as T-cell suppression [25,26,27]. We found that while Ly6G+ cells from both saline- and ECE-treated mice inhibited the proliferation of both CD8+ and CD4+ T cells, a known effect of gMDSCs [26], the Ly6G+ cells from ECE-treated mice were slightly less inhibitory (Figure 3C,D).

**Figure 4:**
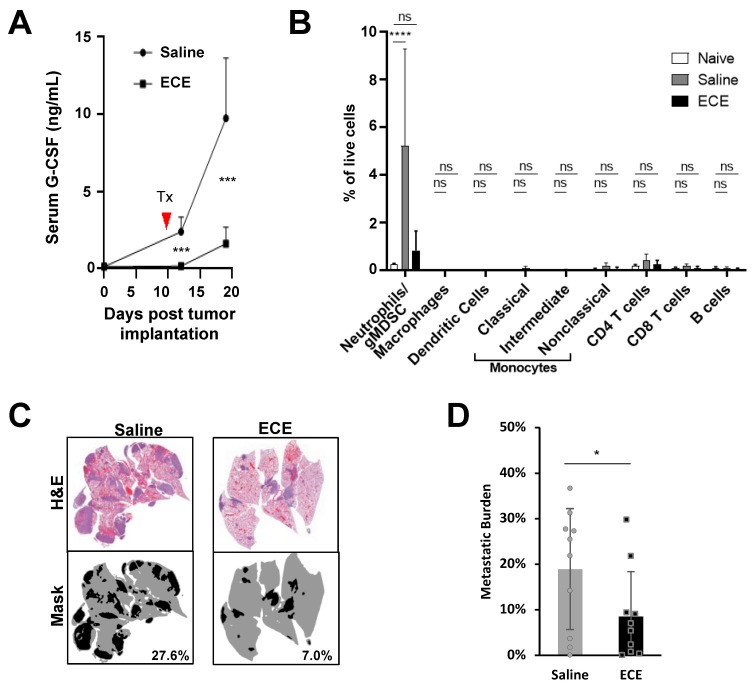
ECE diminishes lung gMDSCs and reduces metastatic growth in 4T1 bearing mice. (**A**) Serum G-CSF levels (*n* = 6) assessed prior to 4T1 tumor implantation and days 2 & 6 following i.t. saline or ECE treatment by ELISA. (**B**) Myeloid and lymphoid immune cell subsets within lungs of 4T1 tumor-bearing mice 7 days following treatment (*n* = 6). (**C**) Representative images of whole lung H&E with mask generated for quantification. (**D**) Metastatic burden of mice after either saline or ECE, *n* = 10. * *p* < 0.05, *** *p* < 0.001, **** *p* < 0.0001.

4T1 tumors injected in the mammary fat pad develop spontaneous pulmonary metastases in almost 100% of mice, a process driven largely by gMDSCs, which migrate to secondary organs (e.g., lungs) where they facilitate the establishment of the pre-metastatic niche [27]. The generation of gMDSCs in the 4T1 TNBC model is promoted by factors secreted by the tumor and host cells, namely G-CSF. Thus, G-CSF plays an instrumental role in 4T1 metastasis, evidenced by the fact that neutralization of G-CSF is capable of dramatically reducing metastasis of this tumor [25,27]. Two days after intra-tumoral ECE monotherapy, we detected serum G-CSF levels similar to that of tumor naïve mice (Figure 4A). G-CSF levels rose slightly over the next 6 days but remained significantly lower than levels found in the serum of saline-treated controls. Correspondingly, we observed decreased numbers of gMDSCs within the tumor (Figure 3A), spleen (Figure 3B), and lungs (Figure 4B) of ECE-treated mice. Consistent with the previously described role of gMDSCs in 4T1 metastasis, we found that lungs from ECE-treated mice possessed significantly fewer metastases compared to saline-treated controls (Figure 4C,D). The ability of ECE to alter the G-CSF/gMDSC axis in the 4T1 model illustrates that local ECE treatment can promote anti-tumor/metastatic effects via systemic immunomodulation of pro-tumorigenic factors and amelioration of immunosuppressive functions by tumor-entrained myeloid cells.

### 2.4. ECE Therapy Sensitizes 4T1 Tumors to Checkpoint Blockade Immunotherapy

**Figure 5:**
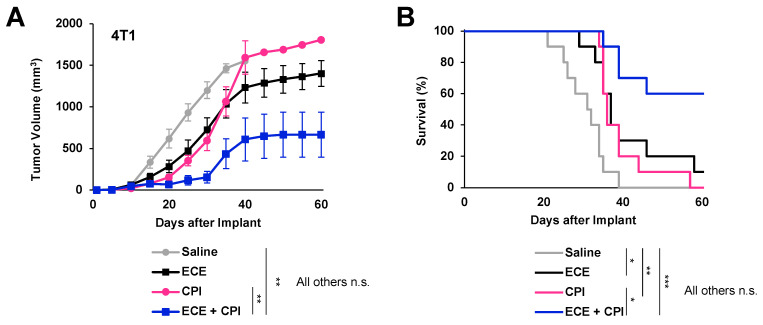
ECE sensitizes 4T1 tumors to checkpoint blockade therapy. (**A**) Average tumor growth and (**B**) Kaplan–Meier survival curves of mice receiving either saline, ECE, CPI, or ECE + CPI, *n* = 10. * *p* < 0.05, ** *p* < 0.01, *** *p* < 0.001.

Our data shows that ECE therapy can reduce both the overall number and suppressive function of gMDSCs in 4T1-bearing mice. We, therefore, hypothesized that this immune environment would be more conducive to T-cell based therapies, such as checkpoint blockade immunotherapy. Mice bearing 4T1 tumors were treated with saline or ECE and then given anti-PD1 + anti-CTLA4 or isotype antibodies. Checkpoint blockade plus intratumoral saline therapy was able to induce a modest antitumor effect; however, all mice succumbed to their cancer by day 60 post tumor implantation. Mice receiving checkpoint blockade plus ECE therapy, on the other hand, displayed a significantly improved antitumor benefit, with most mice surviving at 60 days post tumor implantation (Figure 5A,B). 6 out of 10 mice treated with ECE + CPI had no detectable disease at day 60 of the study, while those treated with CPI and saline all succumbed to large tumor burdens around day 40 of the study. These data demonstrate that ECE therapy can effectively be used in combination with immunotherapies to promote antitumor benefits.

## 3. Discussion

We have previously shown that ECE induces profound necrosis, the effects of which can promote appreciable antitumor benefits against solid tumors when administered intratumorally [7,9]. In this study, we expand upon these findings by showing that intratumoral ECE monotherapy induces immune-mediated changes that facilitate antitumor efficacy in a heterogenous cohort of model breast carcinomas, including the basal-like (4T1) and claudin-low (67NR) subtypes of TNBC. These data highlight ECE treatment as a simple therapeutic strategy that can produce local tumor necrosis while also engendering immunomodulatory effects that can be leveraged when used in combination with other immunotherapeutic strategies.

One of the most striking effects of ECE monotherapy is its ability to provoke systemic immunological changes that reduce immune dysfunction and metastasis. Many solid tumors, including breast carcinomas, co-opt cytokines and cells of the immune system to facilitate their growth and metastasis. Considerable evidence is mounting that tumor-entrained granulocytic cells play a large role in breast cancer progression [28]. Like many immune cells within the myeloid compartment, granulocytic cells can adopt an anti-tumor or pro-tumor phenotype. Polarization towards the pro-tumorigenic phenotype may occur through exposure to TGF-β [29] and results in the generation of gMDSCs (also known as PMN-MDSCs) with profound immunosuppressive capabilities [30]. In 4T1 tumor-bearing mice, tumor- and host cell-derived G-CSF promotes the expansion of gMDSCs [25], and the presence of lung gMDSCs drives the growth of tumor metastases in the lungs [26,27]. Our results show vigorous destruction of the tumor environment by ECE monotherapy is able to dramatically suppress G-CSF production by 4T1 tumors. This outcome promotes system-wide antitumor benefits by diminishing splenic/lung/tumor gMDSC numbers, gMDSC suppressive ability, and 4T1 lung metastases. Interestingly, we and others have found that surgical resection of primary 4T1 tumors does not reduce lung metastases, despite the fact that surgery decreases, but does not eliminate, serum G-CSF levels and gMDSC numbers in the lungs [26,31]. This may be due to the fact that operational stress upregulates a number of immunosuppressive factors (e.g., VEGF, IL-10, TGF-β) that may facilitate establishment of metastatic niches in secondary organs and exacerbate tumor growth [31]. Thus, ECE therapy may have advantages as an adjunct prior to surgical resection of solid tumors for primary ablation without the toxicity of ionizing radiation of chemotherapy.

ECE monotherapy increased T-cell density and enhanced antitumor T-cell responses in poorly metastatic solid breast tumors (i.e., DMBA-induced and 67NR). One possible explanation for this outcome is that ECE treatment potentiates immunogenic cell death. Histologically, ethanol-induced cell death is defined as coagulative necrosis—a form of accidental (non-programmed) cell death. Necrotic cells may be immunogenic, so long as they release damage-associated molecular patterns (DAMPs) that can serve as adjuvants required for the priming of tumor-specific T-cells [17]. On the other hand, we did not observe increased intratumoral T-cell numbers following treatment of metastatic 4T1 tumors with ECE. 4T1 tumors are distinct from DMBA-induced and 67NR breast tumors in that they are enriched in T-cell suppressing gMDSC [27,32]. Our data indicate that gMDSCs make up ~3% of the viable cellular mass of saline-treated 4T1 tumors and ~1% of the viable cellular mass of ECE-treated tumors at 7 days post therapy, while CD8+ T cells make up less than 0.2%. At these ratios, there is likely considerable T-cell suppression by gMDSCs [33,34]. Nevertheless, ECE monotherapy proved capable of significantly reducing the number and suppressive function of tumor-resident and systemic gMDSCs. This, in turn, sensitized tumors to T cell-based checkpoint inhibitor (CPI) immunotherapy with anti-PD1 and anti-CTLA4 antibodies, as we and others have shown [35,36]. These findings have hopeful clinical implications, considering that high counts of circulating gMDSCs are associated with poor prognostic outcomes and higher rates of metastases in breast cancer patients [37,38]. Furthermore, metastatic breast cancer has been shown to have an overall response rate of only ~18% to anti-PD1 and anti-CTLA-4 combination therapy [39]. TNBCs with high PD-L1 expression have higher response rates to anti-PDL1 checkpoint blockade; unfortunately, the majority of TNBC tumors often lack PD-L1 expression and, consequently, poorly respond to checkpoint blockade [40,41]. Therefore, ECE may offer an attractive means to enhance tumor immunogenicity via fulminant necrosis in a minimally invasive, non-ionizing, and low-cost manner.

In conclusion, this study shows that ECE has immunomodulatory properties in non-metastatic and metastatic murine breast cancers. These data have encouraging implications for low- and middle-income countries (LMICs), where mortality rates for breast cancer remain higher due to a lack of healthcare resources. For example, many individuals afflicted with cancer in LMICs do not have access to basic radiation therapy or safe surgery which is the frontline treatment for most non-metastatic breast cancers. Other non-surgical ablative strategies, such as radiofrequency therapy, microwave therapy, cryotherapy, and photodynamic therapy, have appreciable energy demands or lack portability and, therefore, are limited in their utility for LMICs. ECE, on the other hand, does not suffer from these constraints and may provide a much-needed therapeutic option for patients without access to any locoregional therapy.

Moving forward, it will be crucial to determine low-cost therapies that can be used in conjunction with ECE to potentiate treatment effects, as many immunotherapies, such as CPI, remain too expensive for equitable accessible treatment in LMICs. ECE may be easily added to current treatment protocols as ECE is non-ionizing and minimally invasive. For example, the use of ECE with low-dose cyclophosphamide and bicarbonate buffering has recently been shown to produce potent anti-tumor and anti-metastatic effects in TNBC models [12]. Nevertheless, ECE represents a minimally invasive, low-cost, and immune modulating ablative therapy that warrants further investigation.

## 4. Materials and Methods

### 4.1. Tumor Cell Lines

Murine breast cancer cell line 4T1 was purchased from the ATCC. Murine breast cancer cell line 67NR [42] was provided by the Dr. Fred R. Miller laboratory (Karmanos Cancer Institute, Detroit, MI, USA) through Dr. Inna Serganova and Dr. Jason Koucher (Memorial Sloan Kettering Cancer Center, New York, NY, USA). 67NR tumors and metastases were verified with cytokeratin staining, CK8/18, by Dr. Bob Cardiff’s laboratory (University of California Davis, Davis, CA, USA). E0771 cells were provided by Dr. Greg Palmer’s laboratory (Duke University, Durham, NC, USA). Mycoplasma contamination for all cell lines was checked using MycoAlert (Lonza) when frozen cultures were recovered, and cells were not passaged more than 10 times. 67NR and 4T1 cells were cultured in RPMI 1640 (VWR) with 5% FBS, 2 mM L-glutamine, 100 units/mL penicillin, and 100 mg/mL streptomycin. E0771 cells were cultured in RPMI with 5% FBS, 2 mM L-glutamine, 10 mM HEPES, 100 units/mL penicillin, and 100 mg/mL streptomycin.

### 4.2. Murine Mammary Tumor Models

All animal work was approved by the Duke University Institutional Animal Care and Use Committee (IACUC) in compliance with the Guide for the Care and Use of Laboratory Animals published by the US National Institutes of Health (NIH publication 8th edition, revised 2011). Mice were group housed in cages of 5 with standard chow and water ad libitum unless otherwise specified. Mice were enriched with supplemental housing and bedding. Pain was managed with local lidocaine for ablations and mice were euthanized when any humane endpoint was met. 67NR and 4T1 tumors were established in 5–6-week-old female BALB/c mice (Charles River Labs) through injection of 5 × 10^5^ cells in 100 μL of serum-free RPMI 1640 in the 4th mammary fat pad. For checkpoint inhibitor blockade 4T1-bearing mice were given 10 mg/kg anti–PD-1 (BioXcell, clone RMP1-14) and anti–CTLA-4 (BioXcell, clone 9D9) antibodies intraperitoneally on days 12, 15, and 17 after tumor implantation. Mice were euthanized by CO_2_ inhalation and organ removal if tumors exceeded 15 mm in diameter or any other humane endpoint (loss of >15% body weight, failure to groom, self-mutilation, etc.).

### 4.3. Rat DMBA Induced Mammary Tumor Model

Female Sprague Dawley rats (Charles Rivers) were administered a single oral gavage of 50 mg of 7,12-Dimethylbenz[a]anthracene (DMBA) from Thermo Fisher (SKU: AC408181000) dissolved in mineral oil when they reached an age of 50 days. Rats were palpated for tumors twice weekly for 5 months. When a palpable tumor nodule was identified (~1000 mm^3^ in volume), the rat was randomized to receive either ECE or saline injections. After identifying the initial tumor, 200 µL of either saline or ECE was injected directly into the tumor under isoflurane anesthesia for 4 weeks (on study days 0, 7, 14, and 21). On day 30 of the study all tumors were excised. If a tumor reached the tumor burden limit of 5000 mm³ before day 30, the rat was euthanized by CO_2_ inhalation and bilateral thoracotomy and tumors were collected at that time for histology.

### 4.4. Murine Allograft Tumor Growth and Survival

For tumor growth and survival studies in mice, treatment began when tumors were palpable (~50 mm^3^ in volume). Tumor length (L) and width (W) was measured using calipers every 3 days, and volume (V) was calculated using V = (W^2^ × L)/2. Mice were euthanized by CO_2_ inhalation and organ removal if tumors exceeded 15 mm in diameter or any other humane endpoint (loss of >15% body weight, failure to groom, self-mutilation, etc.). Time to a tumor volume of 1500 mm^3^ was used as a surrogate for survival time. If the mouse died or a humane endpoint was met before the maximum tumor burden, the humane endpoint was recorded as an adverse event. If mice made it to 60 days without a tumor or any evidence of a primary tumor, they were euthanized on day 60 to collect lungs for metastatic analysis.

### 4.5. Ethyl-Cellulose Ethanol (ECE) Ablations

Anhydrous ethanol was obtained from Sigma Aldrich (Aldrich, St. Louis, MO, USA; SKU: V001567). USP grade Ethyl-cellulose (Sigma Aldrich, SKU: 1265504) and USP grade lidocaine (Sigma Aldrich, SKU: 1366002) were used for all ablations. Anhydrous ethanol was mixed with 10% *w*/*w* ethylcellulose for the ECE ablations. As a local analgesic, 1% *w*/*w* lidocaine was mixed in to the ECE solution for all ablations. When tumors became first palpable (~50 mm^3^ in volume), mice were anesthetized with inhaled 1–3% isoflurane then ablated with an ECE injection. Mammary tumors were isolated by grasping with tweezers and a 27G half inch needle was used to slowly inject 6 mL/kg (100–150 µL) of ECE by hand. Mice were monitored for adverse events for 1 h after ablation. As a control, mice were injected with sterile phosphate buffered saline (PBS) (Thermo Fisher, Waltham, MA, USA; SKU: 10010023) in the same manner that ECE was injected.

### 4.6. Immunohistochemistry

For CD3 immunohistochemistry, tissues were formalin fixed paraffin embedded by the Duke Substrate core and stained by the Duke Immunohistochemistry core. CD3+ cells were counted in a blinded manner by JIE at 40× in 20 randomly selected fields throughout the tumor. Edges of the tumor on average had higher lymphocytic density and were avoided. Additionally, necrotic regions were avoided in all samples due to high amounts of non-specific staining in necrotic regions. Statistical comparison was performed using Tukey’s HSD test.

### 4.7. Lung Metastases Quantification

After euthanasia, lungs were perfused with 10% neutral buffered formalin and fixed for 24–48 h. Whole lungs were paraffin embedded and stained with hematoxylin and eosin by the Duke Substrate Core. Slides were scanned at 10× and stitched using a Zeiss Axio Imager Widefield Microscope (Light Microscopy Core Facility). Images were compressed 95% and a custom algorithm was used to automatically detect metastatic lesions and total lung volume excluding blood and other tissues. The algorithm’s accuracy was verified by a pathologist using 10 randomly selected raw images and masks. The “metastatic burden” was quantified as the area of metastatic lesions divided by total area of lung tissues.

### 4.8. Flow Cytometry

Flow cytometry was performed on tumors, lungs, and spleens from tumor bearing mice 17 days post tumor implantation. For tumors, a wedge resection ~6 mm in length was harvested and minced into small pieces. Tumors or the left lobe of the lungs were digested in RPMI 1640 (Gibco, Waltham, MA, USA) containing 100 U/mL DNase I (Millipore Sigma, Burlington, MA, USA) and 100 μg/mL Liberase™ (Roche, Basel, Switzerland) and rotated in 5 mL round bottom tubes for 45 min at 37 °C. Digested tumor, digested lung, or spleen were pressed through a 70 μM strainer with the hard end of a syringe and rinsed with 15 mL magnesium/calcium-free PBS (Gibco). Tissue homogenate was centrifuged at 350× *g* for 10 min, supernatant decanted, and washed once with 15 mL magnesium/calcium-free PBS. One-fifth of the tumor or lung homogenate or 10^6^ splenocytes were stained with Zombie Green™ viability dye (BioLegend, San Diego, CA, USA) for 15 min at room temperature, washed once in FACS buffer (PBS + 2% FBS), and then stained with either myeloid or lymphoid panel antibodies.

Myeloid panel: anti-CD45-BV421 (clone 30-F11, BioLegend), anti-Ly6C-BV605 (clone HK1.4, BioLegend), anti-Ly6G-BV785 (clone 1A8, BioLegend), anti-CD11c-PE (clone N418, BioLegend), anti-CD64-PE/Dazzle 594 (clone X54-5/7.1, BioLegend), anti-F4/80-AF700 (clone BM8, BioLegend), anti-CD11b-APC (clone M1/70, BioLegend) anti-MHC II-APC/Fire750 (M5/114.15.2, BioLegend).

Lymphoid panel: anti-B220-BV605 (cloneRA3-6B2, BioLegend), anti-CD8-BV785 (clone 53-6.7, BioLegend), anti-CD3-PE (clone 17A2, BioLegend), anti-CD25-PE/Dazzle 594 (clone PC61, BioLegend), anti-CD19-AF700 (clone 6D5, BioLegend), anti-CD40APC/Fire 750 (cloneGK1.5, BioLegend).

Data were acquired on a CytoFLEX B5-R3-V5 (Beckman Coulter) flow cytometer and analyzed using Kaluza (RRID:SCR_016182, Version 2.1, Beckman Coulter, Indianapolis, IN, USA) software.

### 4.9. G-CSF ELISA

150 μL of blood was collected from mice bearing 4T1 tumors by retro-orbital bleed on days 0, 11, and 19 after orthotopic tumor implantation. After clotting, blood was centrifuged at 2000× *g* for 10 min at 4 °C. Serum supernatant was collected, and G-CSF quantified using ELISA (R&D Systems, Minneapolis, MN, USA) per the manufacturers protocol.

### 4.10. T-Cell Suppression Assay

Spleens were harvested from mice bearing 17-day-old 4T1 tumors or tumor-naïve mice. Following dissociation and red blood cell lysis (Pharm Lyse™, BD Biosciences, Franklin Lakes, NJ, USA), Ly6G+ cells were enriched from spleens of tumor-bearing mice and Ly6G+, CD4+, and CD8+ cells were enriched from spleens of tumor-naïve mice using biotinylated anti-Ly6G antibody (clone 1A8, BioLegend), anti-CD4+ (clone GK1.5, BioLegend), or anti-CD8+ (53-6.7, BioLegend) bound to magnetic MojoSort™ streptavidin beads (BioLegend). CD11b+Ly6C+Ly6G+ granulocytes, CD3+CD4+ T cells, or CD3+CD8+ T cells were >95% pure, based on flow cytometric analysis. CD4+ and CD8+ T cells were labelled with 300 nM CFSE (Thermo Fisher Scientific), neutralized with RPMI 1640 containing 10% FBS, and then resuspended in serum-free AIM V media (Gibco). In a 96-well plate, 3 × 10^5^ T cells were stimulated with anti-CD3/anti-CD28-coated beads (Dynabeads™, Gibco) and 10^5^ Ly6G+ cells, in AIM V media, were added to some wells. Cells were gently pelleted by centrifugation at 100× *g* for 30 s and incubated at 37 °C/5% CO_2_ for 72 h. Cells were then stained with anti-CD3-APC, anti-CD4-APC/Fire750, and anti-CD8-BV785 and proliferating T cells acquired on CytoFLEX B5-R3-V5 (Beckman Coulter, Brea, CA, USA) flow cytometer and analyzed using Kaluza (Beckman Coulter) software.

### 4.11. Statistical Analysis

Statistical analyses were performed in MATLAB using built-in functions. Outliers were detected with a Grubbs Test. No outliers were found to be excluded. A student’s *t* test was used for all comparisons between two groups. Two-tailed ANOVA testing with unequal variance was performed on all groups between each other and controls with a confidence level of 95%. Post hoc multiple comparisons were performed using Tukey’s HSD test. Survival curves were quantified using Kaplan–Meier analysis, and a log-rank test was performed to determine the significance of a *p*-value less than 0.05 with a confidence level of 95%. * *p* < 0.05, ** *p* < 0.01, and *** *p* < 0.001 indicating statistical significance. NS = data are not significantly different.

## 5. Conclusions

ECE is an injectable tumor ablation method that has immunomodulatory properties in TNBC tumors as we show here. In poorly invasive tumors ECE significantly reduced tumor growth and increased local tumor infiltrating lymphocytes. In a metastatic tumor line, a single ECE injection reduced the suppressive capability and amount of gMDSCs in the tumor, spleen, and lungs. The local and systemic changes induced by ECE produced an anti-metastatic response and increased the responsiveness to CPI (anti-CTLA-4 and anti-PD-1). These findings warrant further investigation of ECE and other ablative therapies as immunomodulators for cancer therapy.

## Data Availability

The data presented in this study are available in the figures in the main text and the supplemental figures. Raw data are available on request from the corresponding author.

## Supplementary Materials

The following supporting information can be downloaded at: https://www.mdpi.com/article/10.3390/cancers14194669/s1, Figure S1: Anti-CD3 immunohistochemistry of tumors treated i.t. with saline or ECE; Figure S2: Multiparameter flow cytometry gating strategy.
