# Supplementary material for: Ethanol Ablation Therapy Drives Immune-Mediated Antitumor Effects in Murine Breast Cancer Models
Source: Cancers (Basel). 2022 Sep 25;14(19):4669. doi: 10.3390/cancers14194669 (PMC9564135; doi:10.3390/cancers14194669)
Supplement: Supplementary file 1 [file cancers-14-04669-s001.zip › cancers-1901930-supplementary.pdf]

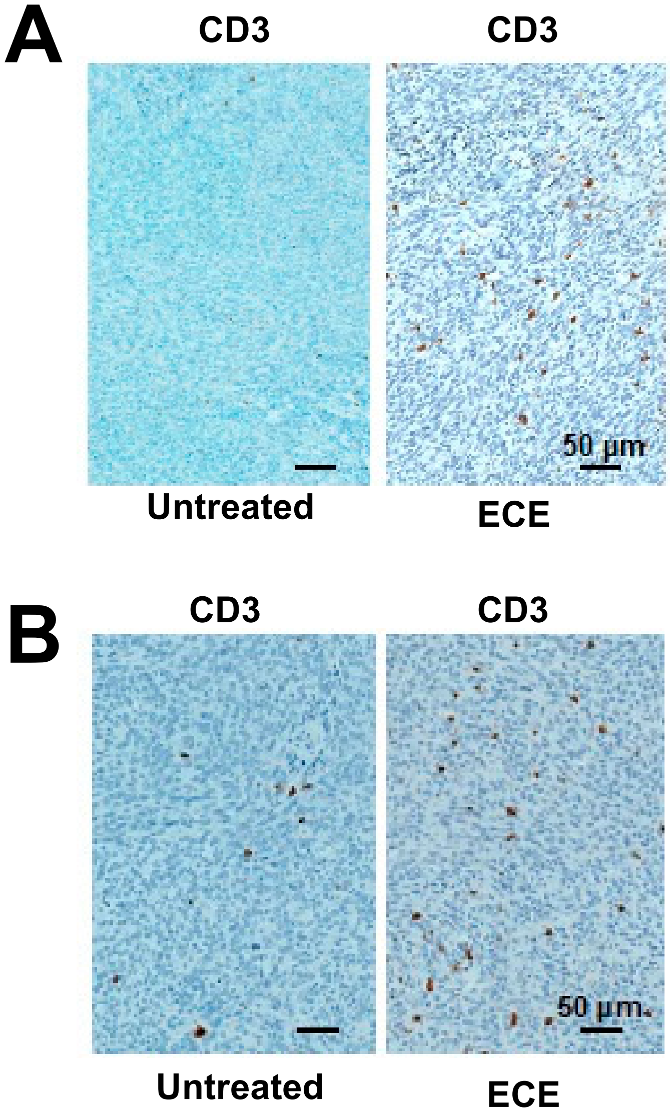

Supplemental Figure S1: Anti-CD3 immunohistochemistry of tumors treated i.t. with saline or ECE. Representative fields of view from (**A**) 67NR and (**B**) 4T1 tumors. i.t. = intratumoral.

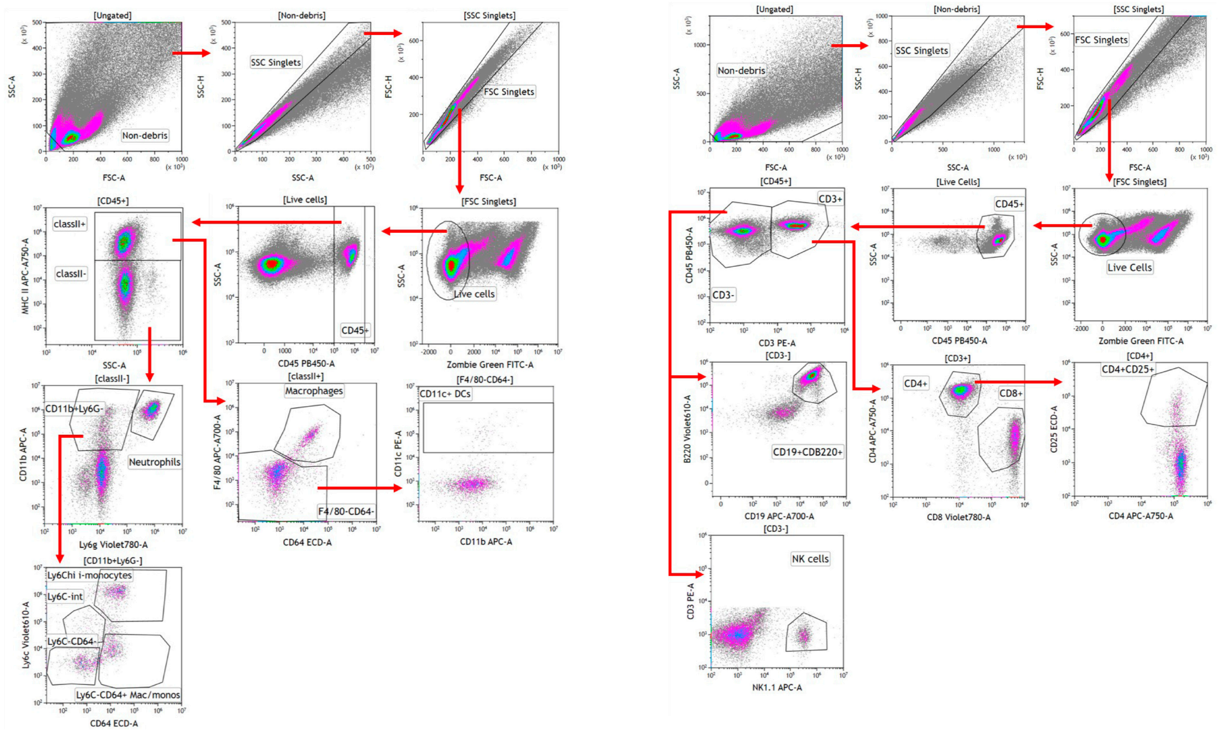

Supplementary Figure S2. Multiparameter flow cytometry gating strategy. Myeloid (left) and lymphoid (right) gating performed on tumor, spleen, and lung tissue. Cells were first gated using SSC-A vs FSC-A to exclude debris followed by SSC-H/FSC-H vs SSC-A/FSC-A to exclude doublets. Zombie green-negative cells delineated viable cells. Major immune cell populations were identified based on their expression of CD45. Within the myeloid population, cells of interest include MHC II+F4/80+CD64+ macrophages, MHC II+CD11c+ dendritic cells, MHC-CD11b+Ly6g+ neutrophils/gMDSCs, CD11b+Ly6c^{HI}MHC II-CD64+ classical/inflammatory monocytes, CD11b+Ly6c^{MOD}MHC II-CD64^{low} intermediate monocytes, and CD11b+Ly6c^{low}MHC II-CD64- nonclassical monocytes. Lymphoid immune cell subsets include CD3+CD8+ T cells, CD3+CD4+ T cells, and CD3-CD19+CD220+ B cells. In some murine models, CD3-NK1.1+ demarcated NK cells; however, this was not used to assess immune cells in BALB/c mice, as these mice lack expression of NK1.1.
